# A SIX1 degradation inducer blocks excessive proliferation of prostate cancer

**DOI:** 10.7150/ijbs.67873

**Published:** 2022-03-14

**Authors:** Yuning Liao, Wenshuang Sun, Zhenlong Shao, Yuan Liu, Xiaoyu Zhong, Yuanfei Deng, Fang Liu, Hongbiao Huang, Jinbao Liu

**Affiliations:** 1 Affiliated Cancer Hospital & institute of Guangzhou Medical University, Guangzhou, Guangdong, 510095, China.; 2 Guangzhou Municipal and Guangdong Provincial Key Laboratory of Protein Modification and Degradation, School of Basic Medical Sciences, Guangzhou Medical University, Guangzhou, Guangdong, 511436, China.; 3 Department of Pathology, First People's Hospital of Foshan, 528000 Foshan, Guangdong, China.

**Keywords:** Prostate cancer, USP1, SIX1, SNS-032, Degradation

## Abstract

Prostate cancer (PC) remains a great medical challenge due to its high incidence and the development of castration resistance in patients treated with androgen deprivation therapy. Deubiquitinases, the enzymes that specifically hydrolyze ubiquitin chains on their substrates, were recently proposed as a serious of critical therapeutic targets for cancer treatment. Our previous study has been reported that the ubiquitin specific peptidase 1 (USP1) functionally acts as a deubiquitinase of sine oculis homeobox homolog 1 (SIX1) and contributes to the proliferation and castration resistance of PC. The stabilization of SIX1 by USP1 partially depends on the status of glucose-regulated protein 75 (GRP75). In this study, we aimed to identify a SIX1 degradation inducer via inhibiting the USP1-SIX1 axis. we screened a range of kinase inhibitors and showed that SNS-032 is the best candidate to trigger the ubiquitinated degradation of SIX1. SNS-032 not only restrains activity of the USP1-SIX1 axis and cell cycle progression, but also results in apoptosis of PC cells. Moreover, the combination of SNS-032 and enzalutamide synergistically induces apoptosis and downregulates expression of USP1, SIX1, and AR/AR-V7 in AR-V7 highly expressed 22Rv1 cells. Overall, our findings may develop a novel and effective strategy to overcome castration resistance in PC for the identification of a SIX1 degradation inducer via targeting the USP1-SIX1 axis.

## Introduction

Prostate cancer (PC) remains a dangerous disease that frequently occurs in old males around the world 1. Currently, androgen deprivation or targeting androgen receptor (AR) therapies are still the clinical mainstay for PC treatment 2. Despite these therapies initially make achievements in most PC patients, the occurrence of castration resistance that developed over time greatly limits their effectiveness on the advanced PC 3. The extremely poor prognosis of castration-resistant PC (CRPC) prompts us to urgently develop novel and substitute therapeutic targets and pharmaceuticals for curing PC.

Protein ubiquitination, a modification controlled by the E1-E2-E3 enzyme cascade and deubiquitinating enzymes (DUBs), is considered as the most common type of post-translational modifications in human cells. The major role of DUBs is to dominate the reversal of protein ubiquitination, a biological process named deubiquitination. It is well accepted that a balance between ubiquitination and deubiquitination is required for maintaining the appropriate protein abundance and physiological status. In contrast, aberrant expression/activity of DUBs may increase the abundance of oncoproteins and cancer drivers, and further contribute to carcinogenesis and tumor progression 4-9. Thus, DUBs have been highlighted in increasingly more studies in oncology and emerged as an attractively new generation of druggable anticancer targets.

Sine oculis homeobox homolog 1 (SIX1) is identified as an evolutionarily conserved critical transcription factor to embryonic development via facilitating the growth of multiple organs 10, 11. During embryonic development, the switch of SIX1 signal is tightly regulated by phosphatase activity of eyes absent (EYA) 12. Generally, the SIX1 signal is inactive post the development of embryo. However, recent reports have been shown that the reactivation of SIX1 signal is observed in various cancers 13-16. Mechanically, SIX1 may drive tumorigenesis via regulating cell cycle progression, glycolysis, and epithelial-to-mesenchymal transition associated factors, such as Cyclin D1, AIB1/HBO1, and vimentin 17-19. Our previous report demonstrated that the expression and stabilization of SIX1 is highly controlled by mitochondria chaperone glucose-regulated protein 75 (GRP75) and ubiquitin specific peptidase 1 (USP1). GRP75 and USP1 can bind to SIX1 and form the “GRP75-USP1-SIX1” complex to prevent degradation of SIX1 from the proteasome, which further boosts the growth and castration resistance of PC 20.

Herein, we intend to screen a SIX1 degradation inducer via targeting the USP1-SIX1 axis in PC. We identify SNS-032, a recently reported antagonist of CDK2/7/9 that exerts anti-tumor activities in multiple cancers 21-24, as an outstanding inducer of SIX1 degradation by downregulating expression of USP1 and triggering K48-linked ubiquitination of SIX1. We show that SNS-032 not only suppresses growth of PC cells, but also enhances sensitivity of CRPC cells to enzalutamide, the second-generation AR antagonist. These findings support our notion that targeting SIX1 degradation is a practicably potent strategy for overcoming castration resistance of PC.

## Materials and Methods

### Materials

A custom-built kinase inhibitor library (partial class of kinase inhibitors selected by authors), SNS-032 (#S1145), Dinaciclib (#S2768), Cycloheximide (#S7418), MG132 (#S2619), Bafilomycin A1 (#S1413), bortezomib (#S1013), and enzalutamide (#S1250) were obtained from Selleckchem (Houston, TX). Antibodies: anti-USP1 (#8033), anti-SIX1 (#16960), anti-Cyclin D1 (#55506), anti-p21 (#2947), anti-p27 (#3686), anti-cleaved Caspase 3 (#9661), anti-FLAG-tag (#8146), anti-Ki-67 (#9449), anti-AR (#5153), and anti-AR-V7 (#19672) were from Cell Signaling Technology (Beverly, MA); anti-GAPDH (#ab181602) were from Abcam (Cambridge, MA).

### Cell culture

All PC cell lines were obtained from the American Type Culture Collection. Short tandem repeat profiling was used to validate cell line identities. 22Rv1 cells were grown in RPMI-1640 (Gibco), while PC3 and DU145 cells were grown in DMEM/F12 (Gibco). Medium was supplemented with FBS at a final concentration of 10% before use. Cells were cultured in an incubator under the standard conditions. Cell passaging and seeding were conducted when the cell coverage reached at 70%-85%.

### Cell proliferation assays

Three assays including clonogenic assay, EdU staining analysis, and cell viability detection were performed to determine the proliferation of PC cells as previously described 4, 25.

### Genetic manipulation assay

The siRNA interfering assay was used for genetic knockdown of CDK1/2/7/9. PC cells were seeded on 60 mm-dishes for 24 h. Transfection mixture was established with RPMI opti-MEM (Gibco), lipofectamine™ RNAiMAX (Invitrogen) and siRNAs. The transfected PC cells were cultured at a final concentration (siRNAs) of 50 nM for 48 h. The sequences of siRNAs targeting CDK1/2/7/9 were listed as below. CDK1 siRNA-1: 5'-GGTTATATCTCATCTTTGA-3'; CDK1 siRNA-2: 5'-GTACTGCAATTCGGGAA AT-3'; CDK2 siRNA-1: 5'-GCACCAAGATCTCAAGAAA-3'; CDK2 siRNA-2: 5'- GGATGTGACCAAGCCAGTA-3'; CDK7 siRNA-1: 5'-CAACCAAATTGTCGCCA TT-3'; CDK7 siRNA-2: 5'-CTTACTAGATCTCATACAA-3'; CDK9 siRNA-1: 5'-GC CAAACGTGGACAACTAT-3'; CDK9 siRNA-2: 5'-ACGAGAAGCTCGCCAAGAT -3'. Plasmid transfection was performed as previously reported 20.

### Co-immunoprecipitation and immunoblot assay

The Dynabeads™ Kit (Invitrogen) was used to detect ubiquitination of protein as previously reported 26. Cell lysates were extracted from SNS-032 or control solvent treated PC cells for 12 h. After protein determination and quantification, 1 mg proteins were used to interact with 1.5 mg dynabeads that coupled with SIX1 antibodies. The protein-dynabead-antibody mixtures were incubated on a rotator at 4 °C for 1 h. Blue SDS loading buffer was added into the mixtures after wash with PBS-T for three times. The SIX1-interacting proteins were then separated using a boiling water bath and centrifugation under 13000 rpm for 5 min, respectively. The ubiquitinated level of SIX1 was detected by further western blot for K48-ubiquitin. The western blot analysis was reported previously 27.

### Immunofluorescence assay

As previously reported 20, cells were seeded on a chamber slide (Thermo Fisher) and treated as indicated figure legends. At the end of cell culture, cells were washed with PBS for three times and fixed with 4% paraformaldehyde. 0.5% Triton-X was then used to permeabilize PC cells for 5 min, followed by the blockade with 5% BSA for 30 min. To visualize the distribution and expression of SIX1, USP1, Cyclin D1, or cleaved Caspase 3, the PC cells were incubated with appropriate primary antibodies at 4 °C overnight. Secondary antibodies for immunofluorescence assay were then used to interact with the primary antibodies for 1 h. Cold PBS was used to wash the cells where needed. The nuclei were visualized by DAPI staining. Finally, images were captured by a confocal microscope.

### PCR assay

As previously reported, total RNAs isolated from PC cells were subjected to the RT-PCR experiment 20. **Table S1** lists the PCR primers for GLUT1, LDHA, HK2, USP1, and SIX1. β-actin was used as an internal control. All PCR experiments were carried out at least three independent replicates.

### Cell cycle assay

PC cells exposed to SNS-032 were digested and resuspended after centrifugation for 5 min. After wash with PBS for thrice, cells were fixed with 2 ml cold 70% ethanol supplemented with 0.5 ml cold PBS at 4 °C overnight. After wash with PBS again, cells were incubated with the staining mixture containing 0.2% Triton X-100, RNase A, and propidium iodide (PI) for 30 min. Flowcytometry was used to analyzed percentage of each cell cycle as previous description 28.

### Apoptosis assay

Cell death analysis was conducted using Annexin V-FITC/PI staining assay as we described before 29, 30. Fluorescence microscopy and Flowcytometry were used to analyzed percentage of the FITC/PI-positive cells.

### Animal study

5- to 6-week-old male nude mice were bred at the animal center of Guangzhou Medical University. After approved by institutional animal care and use committees, 2 × 10^6^ 22Rv1 cells were subcutaneously inoculated on each mouse. After 7 days, the mice bearing 22Rv1 xenografts were randomly divided into 2 groups and exposed to SNS-032 (20 mg/kg/2d, i.p.) or vehicle for 30 days. During the mice breeding, the tumor volume was recorded every 3 days and calculated by a formula of a×a×b×0.5 (a represents the prolate axis, b represents the brachy axis). The body weight of mice was also recorded every 3 days. All mice were sacrificed after CO2 inhalation and cervical dislocation. The tumors were taken out and weighed from subcutaneous tissue. The tumor tissues as well as hepatic/nephridial tissues were fixed with 4% paraformaldehyde, embedded with paraffin, and sliced for further analysis of H&E staining and immunohistochemistry as reported previously 26.

### Data analysis

Data are presented as mean and standard deviation (SD) from at least three repeats. To determine statistical probabilities, unpaired Student's t-tests or one-way ANOVA was conducted where appropriate. Statistical analysis was performed with GraphPad Prism 7.0 and SPSS 16.0. The difference between groups was considered statistically significant when *P* < 0.05.

## Results

### The USP1-SIX1 axis-based drug screening in PC

**Figure 1:**
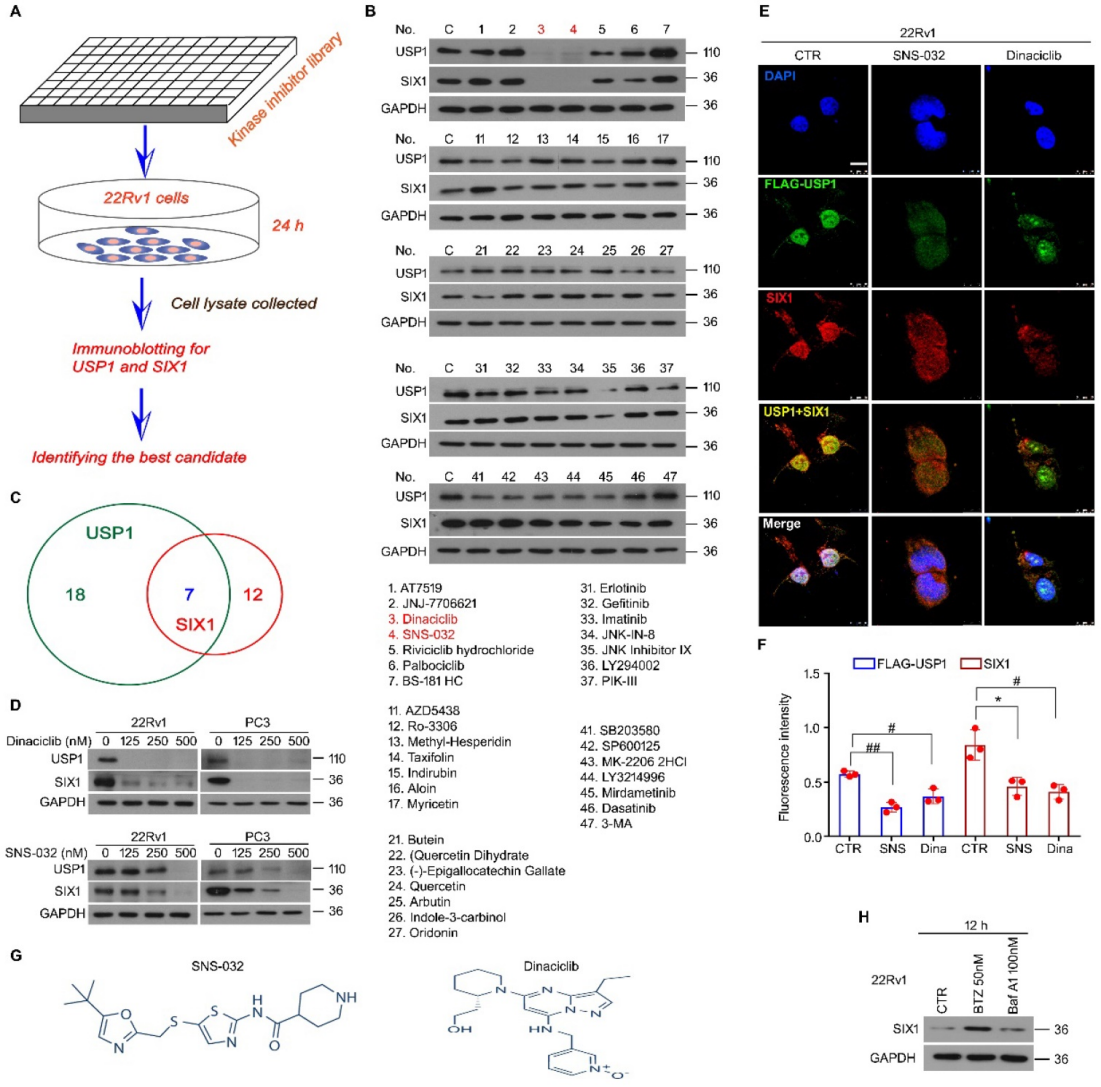
** Kinase inhibitor library screening identifies blockades of the USP1-SIX1 axis. (A)** Schematic illustration of the screening for inhibitor of the USP1-SIX1 axis by using a kinase inhibitor library in PC. **(B)** Immunoblot for USP1 and SIX1 in 22Rv1 cells treated with various kinase inhibitors (all at 0.5 µM) for 24 h. GAPDH was used as a loading control. **(C)** Chemical numbers that can reduce expression of USP1 and SIX1 in B were shown. **(D)** Immunoblot for USP1 and SIX1 in the indicated PC cells exposed to diverse doses of Dinaciclib or SNS-032 for 24 h. **(E)** Confocal microscopy and immunofluorescence assays for SIX1 and FLAG (USP1) in 22Rv1 cells transfected with FLAG-USP1 and treated with Dinaciclib or SNS-032 for 24 h. Scale bar, 10 µm. **(F)** Quantification of the images was shown. (**G**). Structural formula of SNS-032 and Dinaciclib. (**H**). Immunoblot for SIX1 in 22Rv1 cells treated with bortezomib (BTZ) or Bafilomycin A1 (Baf-A1) for 12 h.

We previously reported that USP1 acts as a positive regulator of SIX1 signal at post-translational modification level. The DUB activity of USP1 is required for the protein stability of SIX1, which further mediates proliferation and castration resistance of PC. To explore a potent SIX1 degradation inducer via targeting the USP1-SIX1 axis in CRPC, western blot for USP1 and SIX1 was performed in 22Rv1 cells treated with a series of chemicals (all at 0.5 μM) from a self-established kinase inhibitor library (Fig. 1A). The results showed that there were 7 inhibitors that can downregulate expressions of both USP1 and SIX1. Among these inhibitors, SNS-032 and Dinaciclib were more remarkable in reducing the expressions of USP1 and SIX1 (Fig. 1B-C). We further validated these findings by performing western blot assay in 22Rv1 and PC3 cells exposed to lower doses of SNS-032 and Dinaciclib. We showed that both SNS-032 and Dinaciclib can markedly decreased the expression of USP1 and SIX1 in the lower concentrations (Fig. 1D). Moreover, our immunofluorescence assay showed that SNS-032 and Dinaciclib can also decrease expressions of USP1 and SIX1 in 22Rv1 cells transfected with exogenous FLAG-USP1 plasmids (Fig. 1E-F). Together, the findings indicate that SNS-032 and Dinaciclib are potent inhibitors in reducing protein levels of USP1 and SIX1. As shown in Fig. 1G, Dinaciclib is a pyrazolo[1,5-a]pyrimidine, while the N-(5-{[(5-tert-butyl-1,3-oxazol-2-yl)methyl]sulfanyl}-1,3-thiazol-2-yl)piperidine-4-carboxamide (SNS-032) is a secondary carboxamide resulting from the formal condensation of the carboxy group of piperidine-4-carboxylic acid with the amino group of 5-{[(5-tert-butyl-1,3-oxazol-2-yl)methyl]sulfanyl}-1,3-thiazol-2-amine. We next wondered whether SIX1 may be degraded by proteasome or/and lysosome. The immunoblot showed that protein level of SIX1 was markedly upregulated by bortezomib (an inhibitor of proteasome, BTZ), but not Bafilomycin A1 (an inhibitor of lysosome) (Fig. 1H), indicating that ubiquitin-proteasome system, but not lysosome, mediates SIX1 degradation.

### SNS-032 stood out as a SIX1 degradation inducer

**Figure 2:**
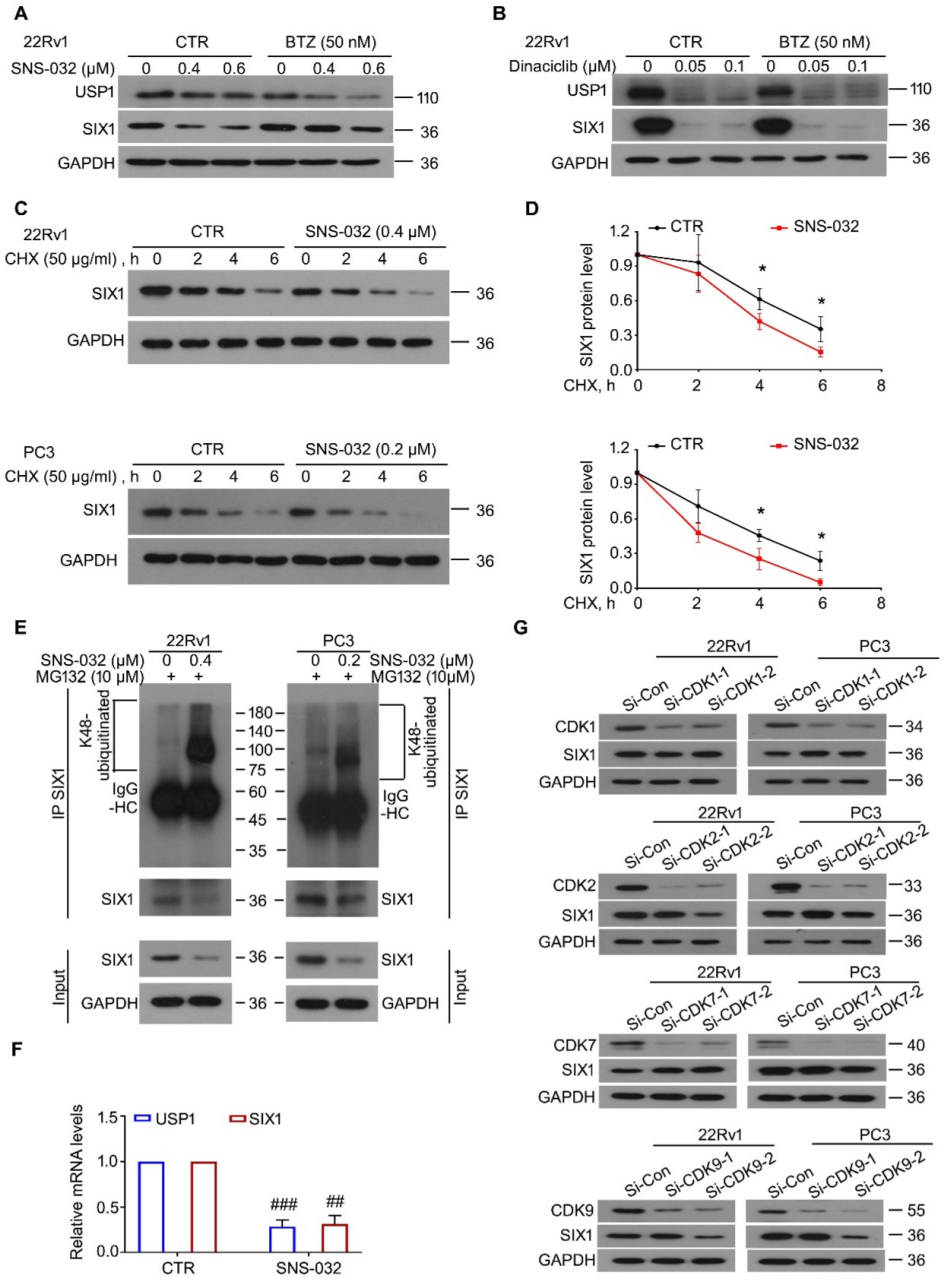
** SNS-032 induces SIX1 degradation through the proteasome. (A)** 22Rv1 cells were exposed to SNS-032, and (**B**) Dinaciclib, in the presence or absence of bortezomib (BTZ) for 12 h, followed by immunoblot for USP1 and SIX1. **(C)** CHX chasing assay was performed in PC cells. **(D)** The curves of SIX1 degradation in C were shown. Mean±SD (n=3), *P<0.05. **(E)** Co-IP/immunoblot assay was performed in PC cells exposed to SNS-032 for 12 h. MG132 was used to accumulate ubiquitinated proteins for 6 h before cell harvest. **(F)** RT-PCR assay for USP1 and SIX1 was performed in 22Rv1 cells treated with SNS-032 for 3 h. Mean±SD (n=3), ^##^*P<*0.001, ^###^*P<*0.0001. **(G)** Immunoblot assay using the indicated antibodies was performed in PC cells transfected with CDK1/2/7/9 siRNAs or control siRNAs for 48 h.

To further determine whether SNS-032 and Dinaciclib downregulated SIX1 via inducing the proteasomal degradation of SIX1, the BTZ rescue experiments post SNS-032 or Dinaciclib treatment were performed. The results showed that BTZ remarkably reversed the SIX1 reduction induced by SNS-032, but not Dinaciclib, in 22Rv1 cells (Fig. 2A-B). These findings indicate that SNS-032, but not Dinaciclib, is the best candidate of SIX1 degradation inducer. Of note, BTZ did not reverse the USP1 reduction caused by SNS-032 or Dinaciclib treatment, which may due to the different half-life of USP1 or alteration at mRNA level. Next, the CHX-tracking analysis was conducted in 22Rv1 and PC3 cells exposed to SNS-032. Our western blot assay and degradation curves showed that SNS-032 notably accelerated the degradation of SIX1 and shortened its half-life (Fig. 2C-D). To examine whether the SNS-032-induced degradation of SIX1 is mediated by ubiquitination, co-IP and immunoblot analysis were conducted in PC cells post SNS-032 treatment. As a result, the K48-linked ubiquitination of SIX1 was accumulated by SNS-032 (Fig. 2E). Collectively, these findings demonstrate that SNS-032 is a SIX1 degradation inducer which boosts the ubiquitination level of SIX1 via downregulating USP1. Moreover, we determined the mRNA alteration of USP1 and SIX1 in 22Rv1 cells post SNS-032 treatment using RT-PCR assay. We showed that mRNA levels of USP1 and SIX1 were both decreased by SNS-032 (Fig. 2F), which may due to the inhibition of RNA polymerase, a previously reported effect of SNS-032 24. These results indicate that SNS-032 reduces SIX1 expression by inducing SIX1 degradation and suppressing SIX1 transcription.

Because SNS-032 is previously characterized as an inhibitor of CDK2/7/9, we next wondered whether CDKs were involved in the modulation of SIX1 degradation. Our immunoblot analysis showed that knockdown of CDK1/2/7/9 (by two pairs of siRNAs for each gene) failed to conformably affect the protein level of SIX1 in both PC cells (Fig. 2G), suggesting that SNS-032 induces degradation of SIX1 independent of CDK1/2/7/9.

### The SIX1 degradation inducer suppressed proliferation of PC cells

**Figure 3:**
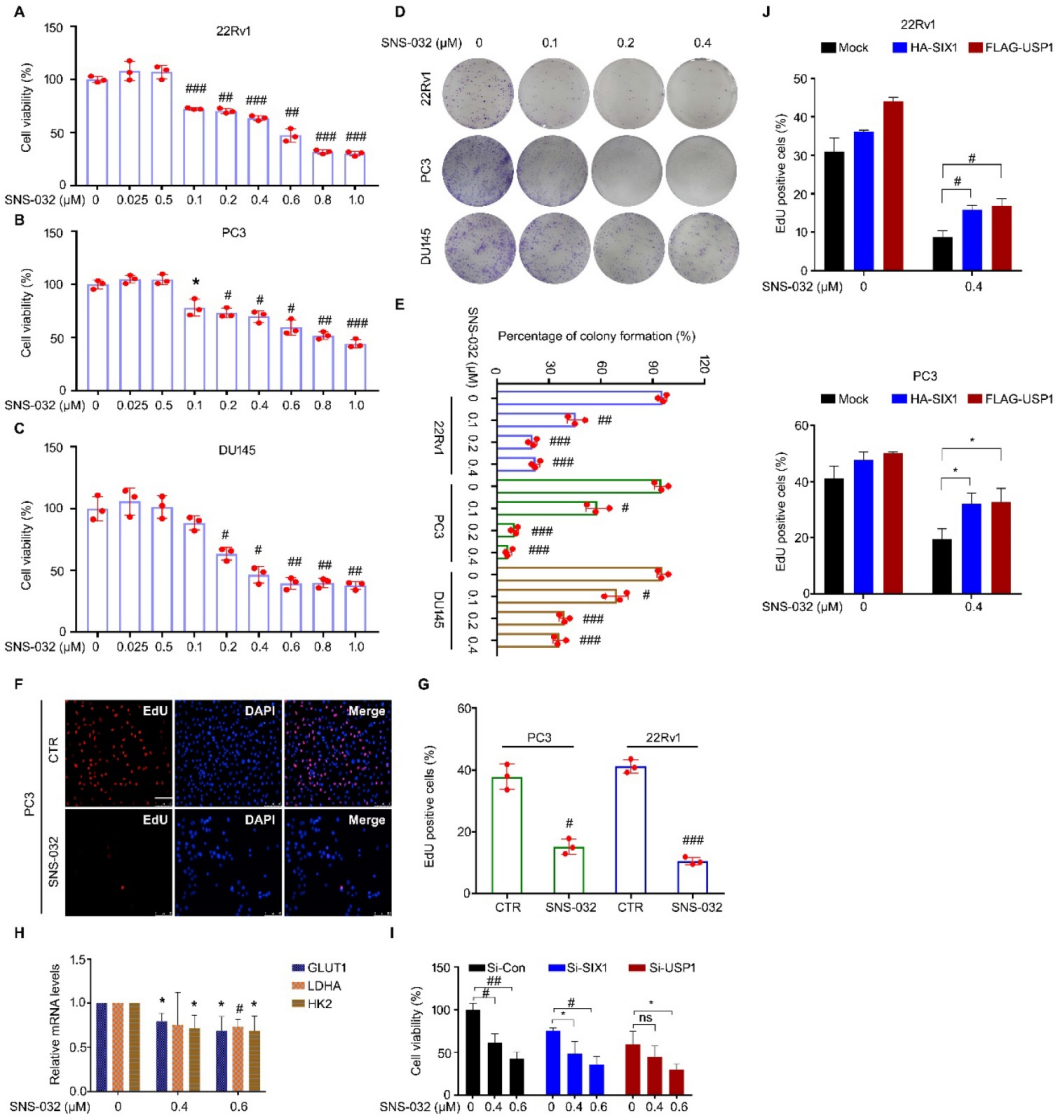
** SIX1 degradation inducer suppresses growth of PC cells. (A-C)** Cell viability of the indicated PC cells exposed to various doses of SNS-032 for 24 h. **(D)** Colony formation assay was performed in the indicated PC cells exposed to SNS-032 for 2 weeks. **(E)** Quantification of relative colony formation in D was shown. **(F)** EdU staining assay was performed in PC cells exposed to SNS-032 for 24 h. Scale bar, 100 µm. **(G)** Quantification of EdU positive cells in F was shown. The above data were presented as mean±SD (n=3), ^*^P<0.05, ^#^*P<*0.01, ^##^*P<*0.001, ^###^*P<*0.0001. **(H)** RT-PCR assay for GLUT1, LDHA, and HK2 was performed in 22Rv1 cells treated with SNS-032 for 12 h. Mean±SD (n=3), ^*^P<0.05, ^#^*P<*0.01. (I) Cell viability assay was performed in 22Rv1 cells treated with si-SIX1, si-USP1, or control siRNA for 24 h, and followed by the treatment of SNS-032 for 48 h. Mean±SD (n=3), ^*^P<0.05, ^#^*P<*0.01, ^##^*P<*0.001. **(J)** EdU staining assay was performed in the indicated cells treated with HA-SIX1, FLAG-USP1, or control plasmids for 24 h, and followed by the treatment of SNS-032 for 48 h. Mean±SD (n=3), ^*^P<0.05, ^#^*P<*0.01.

To address whether SNS-032 suppressed proliferation of CRPC cells after inhibition of the USP1-SIX1 axis and the induction of SIX1 degradation, multiple assays were performed in PC cells. Firstly, cell viability was determined using MTS assay in 22Rv1, PC3, and DU145 cells for 24 h. We showed that the cell viability of these cell lines was significantly reduced post the administration of SNS-032 (Fig. 3A-C). Additionally, the colony formation analysis displayed that the percentage of colony formation was decreased in the three PC cell lines after SNS-032 treatment (Fig. 3D-E), indicating that SNS-032 can suppress the PC proliferation at intermediate stage. Moreover, we determined the ability of DNA replication and synthesis using EdU staining assay in 22Rv1 and PC3 cells. Our data displayed that the percentage of PC cells at replication and synthesis stage (EdU positive cells) was significantly reduced post the treatment of SNS-032 (Fig. 3F-G).

Because SIX1 has been well-characterized as a key regulator of glycolysis, we further investigated whether SNS-032 may alter related glycolytic gene expression using RT-PCR assay. We found that SNS-032 significantly decreased the SIX1-controlled glycolytic gene expression, including GLUT1, LDHA, and HK2 (Fig. 3H), which further supports that SNS-032 inhibits SIX1 signaling. To further elucidate whether the USP1-SIX1 axis may be involved in the SNS-032-induced proliferation suppression, cell viability assay was performed in PC cells treated with SNS-032 with or without si-SIX1 or si-USP1. The results showed that PC cells were less sensitive to SNS-032 in the SIX1-knockdown and USP1-knockdown groups, compared with that in the control group (Fig. 3I). Moreover, our EdU staining analysis showed that overexpression of HA-SIX1 or FLAG-USP1 by transfecting plasmids partially reversed the SNS-032-induced proliferation blockade in PC cells (Fig. 3J), indicating that the USP1-SIX1 axis is an important target of SNS-032 for inhibiting PC. Together, these findings collectively demonstrate that the SIX1 degradation inducer, SNS-032, potently inhibited CRPC proliferation *in vitro*.

### Cell cycle and apoptosis were involved in the proliferation suppression triggered by SIX1 degradation inducer

**Figure 4:**
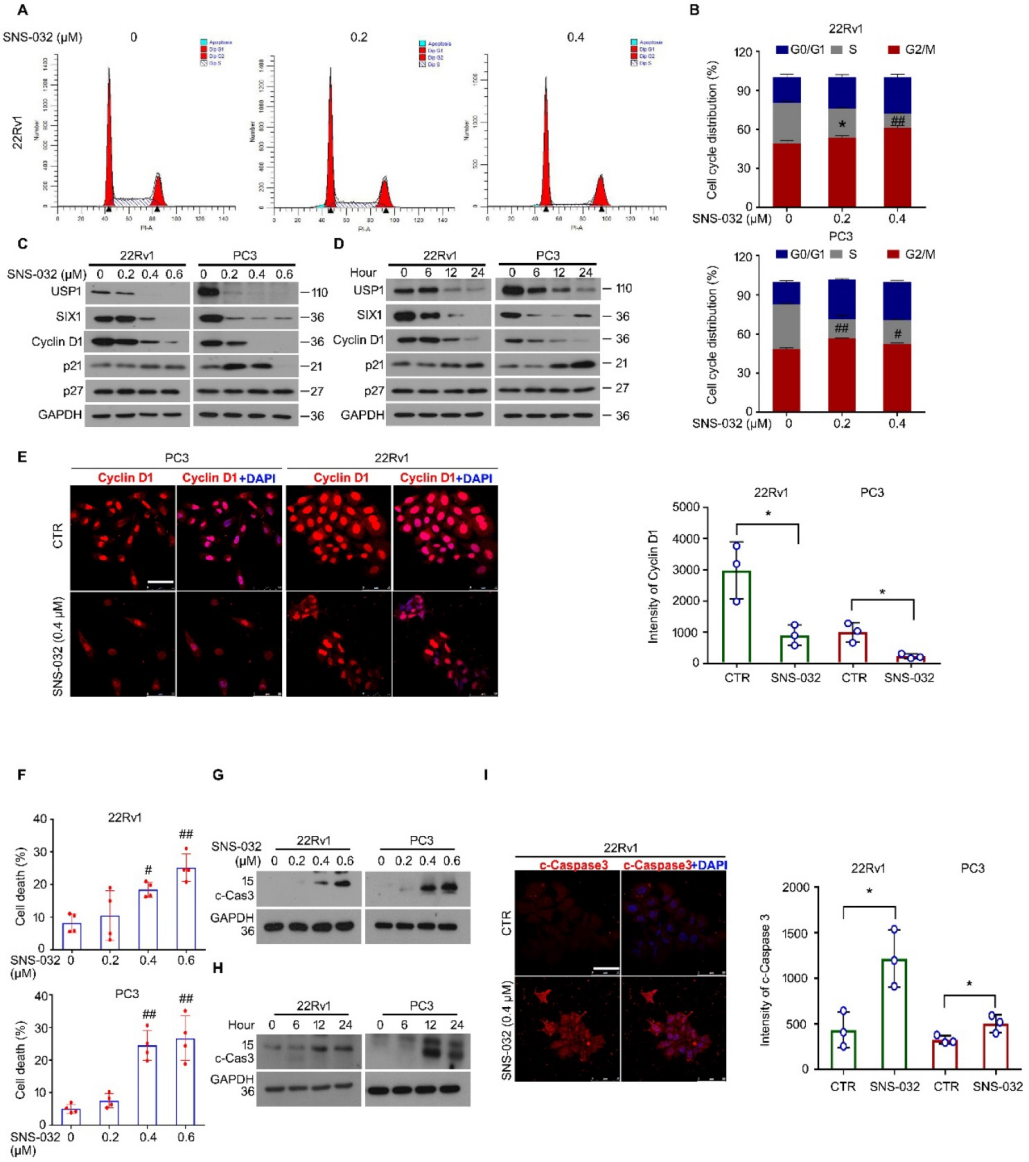
** SIX1 degradation inducer results in G0/G1 phase arrest and apoptosis in PC cells. (A)** Cell cycle assay was performed in PC cells exposed to SNS-032 for 24 h. Representative images of 22Rv1 cells were shown. **(B)** Quantification of cell cycle distributions. Mean±SD (n=3), ^*^P<0.05, ^#^*P<*0.01, ^##^*P<*0.001. **(C)** Immunoblot assay using USP1, SIX1, and Cyclin D1 antibodies was performed in PC cells exposed to SNS-032 for 24 h, and **(D)** various length of time at 10 µM. **(E)** Confocal microscopy/immunofluorescence assays for Cyclin D1 in PC cells. Scale bar, 50 µm (Left). Quantification of the images were shown. Mean±SD (n=3), ^*^P<0.05 (Right). **(F)** Flowcytometry assay was performed in PC cells exposed to SNS-032 for 24 h. Cells were stained with annexin V-FITC and PI. Quantification of cell death was shown. Mean±SD (n=4), ^#^*P<*0.01, ^##^*P<*0.001. **(G)** Immunoblot assay using cleaved-Caspase 3 antibodies was performed in PC cells exposed to SNS-032 for 24 h, and **(H)** various length of time at 10 µM. **(I)** Confocal microscopy/immunofluorescence assays for cleaved-Caspase 3 in 22Rv1 cells. Scale bar, 50 µm (Left). Quantification of the images were shown. Mean±SD (n=3), ^*^P<0.05 (Right).

It is well known that infinite proliferation caused by aberrant cell cycle progression is a basic hallmark of cancer. To further explore whether the cell cycle alteration may mediate proliferation suppression triggered by the SIX1 degradation inducer, cell cycle was determined by flowcytometry analysis in 22Rv1 and PC3 cells post the administration of SNS-032 or vehicle. The results showed that the percentage of G0/G1 phase was elevated, while the percentage of S phase was decreased, in two PC cell lines (Fig. 4A-B). Additionally, the immunoblot analysis displayed that the expression of USP1, SIX1, and Cyclin D1 (a well-defined downstream signal of SIX1 and cell cycle regulator drives the cycle transition from G0/G1 to S phase) were reduced in these PC cells. Meanwhile, we showed that the cyclin-dependent kinase inhibitors, p21, but not p27, was increased by SNS-032 within certain limitations (Fig. 4C-D). Furthermore, the immunofluorescence results also showed that the Cyclin D1 expression was notably inhibited in most PC cells (Fig. 4E). These data collectively indicate that the cell cycle suppression is involved in growth arrest induced by the SIX1 degradation inducer in CRPC cells.

Apoptosis escape is another hallmark of cancer. We next wondered whether the SIX1 degradation inducer may lead to apoptosis in PC cells. Apoptosis was determined by flowcytometry analysis in PC cells stained with PI and annexin V-FITC. We found that the administration of SNS-032 markedly triggered apoptosis in PC cells (Fig. S1A and Fig. 4F). In addition, our western blot and immunofluorescence results showed that expression of cleaved Caspase 3 (the key effector which induces apoptosis) was notably induced in 22Rv1 and PC cells post the exposure of SNS-032 (Fig. 4G-I and Fig. S1B). Moreover, we wondered whether the USP1-SIX1 axis may regulate apoptosis in PC. Apoptosis assay was performed in 22Rv1 cells by the knockdown of USP1 and SIX1 for 2 days. The results displayed that the knockdown of USP1 and SIX1 failed to trigger apoptosis in PC cells (Fig. S1C-D). Taken together, these results demonstrate that the SIX1 degradation inducer, SNS-032, may result in apoptosis which is independent of the USP1-SIX1 axis in CRPC cells.

### The SIX1 degradation inducer inhibited PC growth in mice model

**Figure 5:**
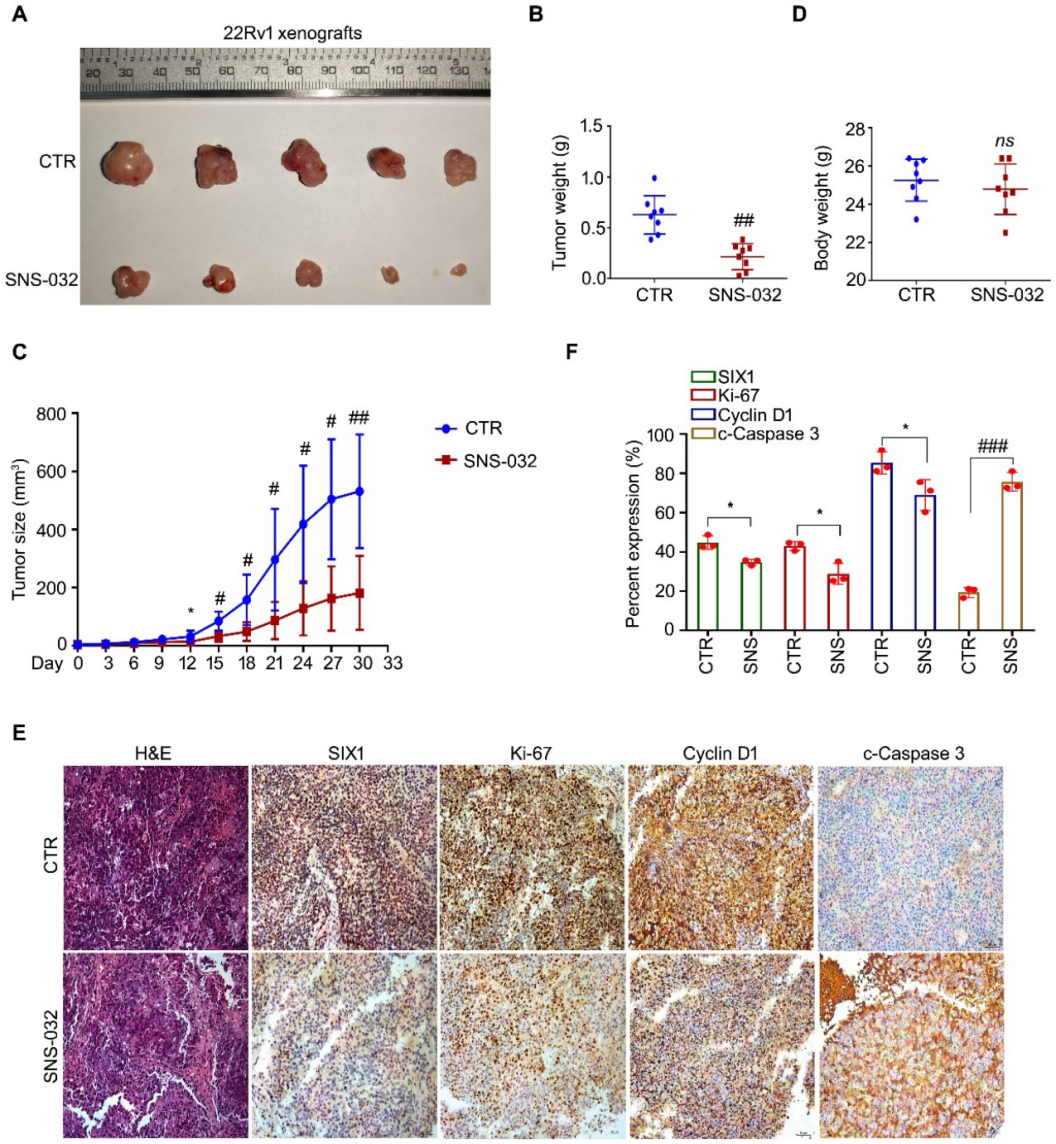
** SIX1 degradation inducer inhibits PC growth in nude mice. (A-D)** 22Rv1 xenografts were established in nude mice and treated with SNS-032 (20 mg/kg/2d, i.p.) or vehicle for 30 days. Tumor images, tumor weights, tumor sizes, and body weights were shown. Mean±SD (n=8), ^*^P<0.05, ^#^*P<*0.01, ^##^*P<*0.001. **(E)** H&E staining and immunohistochemistry assay using SIX1, Ki-67, Cyclin D1 and cleaced-Caspase 3 antibodies were performed in xenograft tissues. Representative images were shown. **(F)** Quantification of the immunohistochemistry images were shown. Mean±SD (n=3). ^*^P<0.05, ^###^*P<*0.0001.

To address the question that whether the SIX1 degradation inducer may display an anti-CRPC activity *in vivo*, 22Rv1 cells were subcutaneously injected and transplanted in BALB/c nude mice. After 1 week, the nude mice bearing 22Rv1 xenografts were randomly separated into 2 groups and treated with SNS-032 or vehicle for 30 days. Our data showed that the volume and weight of tumor, but not body weight of mice, were significantly decreased post the exposure of SNS-032 (Fig. 5A-D). To further determine the key protein alterations in tumor tissues, H&E staining and immunohistochemistry analysis was conducted. Our results displayed that the expressions of SIX1, Cyclin D1, and Ki-67 (a well-accepted proliferation marker) in tissues were notably reduced by the administration of SNS-032, while the expression of c-Caspase 3 (the activated form of Caspase 3) was increased in the treated group (Fig. 5E-F), indicating that SNS-032 can also trigger SIX1 downregulation, cell cycle arrest, and apoptosis in HCC xenografts established on mice models. Together, these data suggest that the SIX1 degradation inducer restrains the proliferation of CRPC *in vivo*.

### The SIX1 degradation inducer re-sensitized CRPC cells to enzalutamide

**Figure 6:**
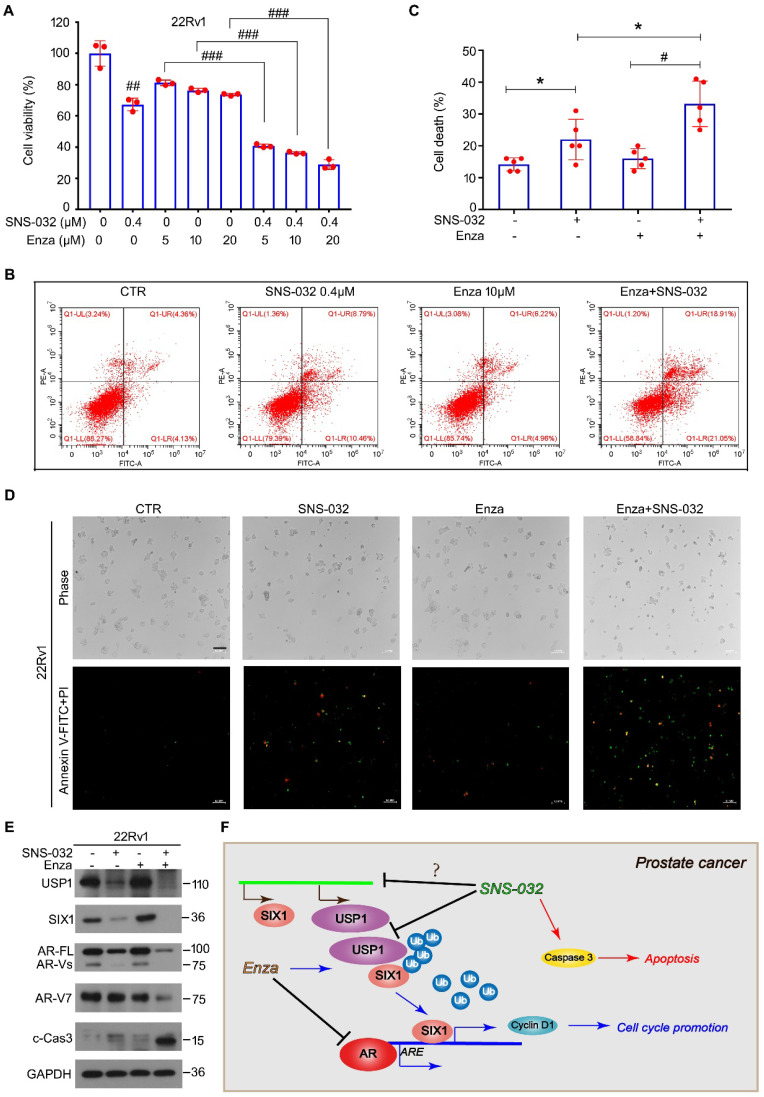
** Sensitivity enhancing of CRPC cells to enzalutamide by SNS-032. (A)** Cell viability of 22Rv1 cells exposed to enzalutamide with or without SNS-032 for 24 h. **(B)** Flowcytometry assay of 22Rv1 cells exposed to enzalutamide with or without SNS-032 for 24 h. Cells were stained with annexin V-FITC and PI. **(C)** Quantification of B was shown. **(D)** Inverted fluorescence microscopy assay was performed in 22Rv1 cells exposed to enzalutamide with or without SNS-032 for 24 h. Cells were stained with annexin V-FITC (green) and PI (red). Scale bar, 100 µm. **(E)** Immunoblot for USP1, SIX1, AR, AR-V7, and cleaved-Caspase 3 in PC cells exposed to enzalutamide with or without SNS-032 for 24 h. **(F)** A proposed mechanism by which SNS-032 induces SIX1 degradation and overcomes castration resistance in PC.

In previous study, we showed that enzalutamide, an AR antagonist, potentially upregulates the protein expression of SIX1 via inhibiting SIX1 degradation, which is related to the occurrence of castration resistance. We therefore wondered whether the responsiveness of CRPC cells to the AR antagonist could be restored by the SIX1 degradation inducer. Thus, cell viability assay was firstly determined in AR/AR-V7 positive 22Rv1 cells treated with enzalutamide and/or SNS-032. Our data showed that the cell viability suppression induced by the combination of enzalutamide and the SIX1 degradation inducer was more remarkable than that of single drug treatment (Fig. 6A). In addition, analysis of apoptosis using flowcytometry was performed. We showed that the combination of enzalutamide and the SIX1 degradation inducer synergistically triggered apoptosis in 22Rv1 cells (Fig. 6B-C). Our morphological analysis on apoptosis performed in 22Rv1 cells was highly consistent with the flowcytometry findings (Fig. 6D). Importantly, the immunoblot analysis further displayed that the combination of enzalutamide and SNS-032 notably reduced expressions of USP1, SIX1, AR and AR-V7 (Fig. 6E), which are key players for the growth and castration resistance of PC. Together, our findings indicate that the SIX1 degradation inducer can re-sensitize CRPC cells to anti-AR therapy.

## Discussion

Targeting degradation of the oncoproteins or cancer drivers is a research hotspot as well as an attractive anti-cancer strategy in current medicine 31. In this study, we found that SNS-032, a chemical that previously identified as an inhibitor of CDK2/7/9, is a SIX1 degradation inducer which displays a remarkable capacity in the induction of SIX1 ubiquitination and degradation via reducing the expression of USP1 in PC cells. The SIX1 degradation inducer can repress the proliferation of CRPC cells and restore sensitivity of the CRPC cells to enzalutamide exposure via targeting the USP1-SIX1-Cyclin D1 axis and triggering apoptosis. Discovery of the SIX1 degradation inducer not only provides a potential tool to explore the regulatory mechanisms of SIX1 signal, but also proposes a previously undefined strategy for PC treatment.

There are about 1,414,259 new cases and 375,304 cancer deaths of PC based on the Global Cancer Statistics in 2020 1, which suggests that PC remains a common cancer with poor prognosis in male. Compared to the other cancers, the molecular mechanisms of occurrence and progression of PC have been better elucidated. It is well accepted that androgen receptor (AR) is the most critical driver responsible for the advancement of PC, according to abundant experimental evidence and the clinical observations that blockade of the AR signal is effective to most cases. However, most patients with PC eventually developed castration resistance to the therapies through targeting the AR signal within 2-3 years, although they initially sensitive to these therapies 32. Unfortunately, the regulatory mechanisms underlie CRPC development remains elusive. Various studies have been demonstrated that reactivation of AR that resulted from the genetic mutation and amplification, increased stability, and expressions of splice variants, may be the major mechanism that contributes to the progress from the androgen-dependent PC to CRPC 4, 26, 33-35. Our previous study demonstrated a novel mechanism that SIX1 signal can also contribute to the progression of CRPC 20. Our current study demonstrated that the SIX1 degradation inducer can elevate the responsiveness of CRPC cells to the anti-AR therapy, consistently supporting that SIX1 is a druggable target for overcoming CRPC.

Recently, selective degradation of the oncogenic drivers has become a promising avenue for the administration of PC. For example, we have previously identified a novel AR-V7 degrader, rutaecarpine, which preferentially induces formation of the GRP78-SIAH2-AR-V7 complex to promote ubiquitination and degradation of AR-V7 in CRPC cells. The rutaecarpine-induced GRP78-dependent degradation of AR-V7 exerts anti-CRPC activity primarily through suppressing cell cycle progression and restoring the responsiveness of cells to enzalutamide 26. In addition, inhibition of proteasomal USP14 with IU1 or auranofin notably results in the increase of AR ubiquitination and degradation 4, 36. Liu, C, et al. showed that targeting the HSP70/STUB1 with ARVib can potently trigger AR/AR-V7 degradation, thereby suppressing proliferation and progression of CRPC 37. Moreover, other groups showed that the application of proteolysis-targeting chimera (PROTAC) technique designed for the degradation of AR 38, AR-V7 39, and BET family proteins 40 displays favorable anti-CRPC activities. Unlike these findings, we here identify SNS-032 as a degradation inducer of SIX1, a new recognized oncogenic driver of CRPC, and update the knowledge and approach to CRPC treatment.

Highly dysregulation of cell cycle process and apoptosis resistance are two basic features contribute to malignant progression of cancer. Numerous studies have been developed a serious of therapeutic targets as well as chemicals for cancer treatment via interfering these features 41, 42. In this study, we found that the SIX1 degradation inducer resulted in the Cyclin D1-dependent G0/G1 phase arrest. Thus, these findings are in accordance with our previous report that Cyclin D1 is a downstream regulator of the USP1-SIX1 axis responsible for the phase transition from the G0/G1 to S phase, demonstrating that Cyclin D1-driven cell cycle progression is a critical target for the SIX1 degradation inducer against castration resistance of PC. In addition, we found that the SIX1 degradation inducer can trigger the activation of Caspase 3 and typical apoptosis that was independent of the USP1-SIX1 axis because the genetic silence of either USP1 or SIX1 failed to induce apoptosis in PC cells. Moreover, the combination of enzalutamide and the SIX1 degradation inducer synergistically boosted apoptosis in PC cells. Overall, our findings indicate that the SIX1 degradation inducer, SNS-032, potentially arrests the USP1-SIX1-Cyclin D1-driven cell cycle progression and the USP1-SIX1 axis-independent apoptosis required for proliferation suppression of CRPC cells. Despite this study does not investigate on how SNS-032 triggers apoptosis, SNS-032 exerts apoptosis induction effect in many cell types via inhibition of CDK1/2/7/9. Therefore, SNS-032-induced CDKs inhibition may not lead to the arrest of cell cycle progression, but result in the USP1-SIX1 axis-independent apoptosis.

Although this study has addressed the questions of our greatest concern, there are still many research expectations to improve the values of clinical translation via targeting SIX1 degradation in future. Firstly, drug modifications on SNS-032 could be explored to optimize the anti-CRPC effects and the selectivity on SIX1 degradation. In addition, other chemical libraries, such as natural products and FDA-approved drug library, could be included to the screening on SIX1 degradation via targeting the USP1-SIX1 axis. Furthermore, a chemical screening on the SIX1 degradation via enhancing the activity of APC (Cdh1), an E3 ligase that has been identified as a key player to induce ubiquitination of SIX1 43, could also be explored in the next investigations. Indeed, there are two major shortages of this study. On one hand, the underlying mechanisms responsible for the downregulation of AR/AR-V7 post the combination of enzalutamide and the SIX1 degradation inducer were elusive in CRPC cells. On the other hand, due to the technical barrier, this study evaluated the *in vivo* anti-CRPC effects of the SIX1 degradation inducer by the establishment of subcutaneous tumor xenograft, but not orthotopic transplantation in the prostate.

In summary, this study identifies a SIX1 degradation inducer that potently inhibits CRPC cell proliferation via targeting the USP1-SIX1 axis and triggering apoptosis of PC cells *in vitro* and *in vivo*, and further provides a novel strategy with the combination of enzalutamide and the SIX1 degradation inducer for overcoming the progression of castration resistance during PC treatment (Fig. 6F).

## Supplementary Material

Supplementary figure and table.
