# Supplementary material for: A SIX1 degradation inducer blocks excessive proliferation of prostate cancer
Source: Int J Biol Sci. 2022 Mar 14;18(6):2439–51. doi: 10.7150/ijbs.67873 (PMC8990483; doi:10.7150/ijbs.67873)
Supplement: Supplementary file 1 — Supplementary figure and table. [file ijbsv18p2439s1.pdf]

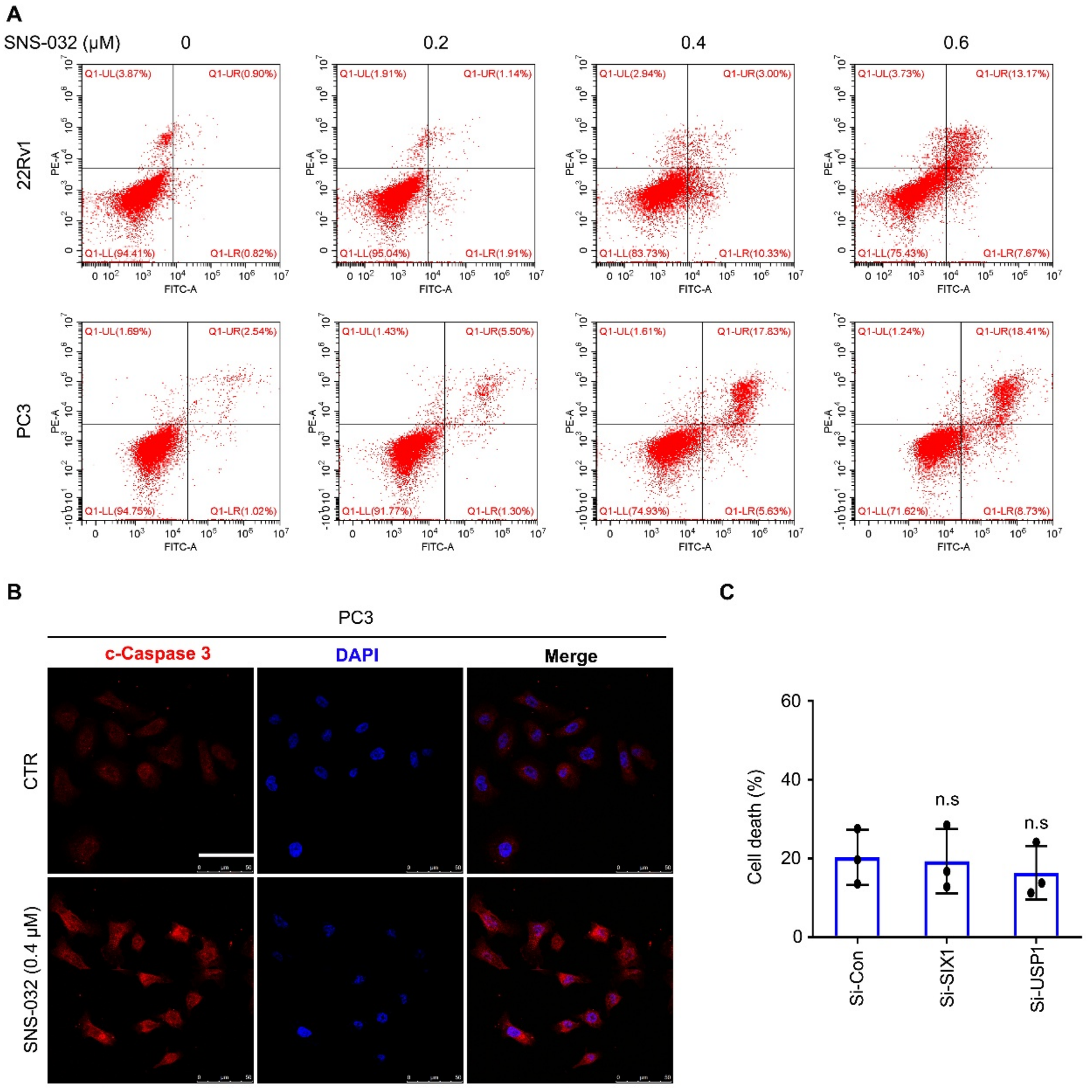

Figure S1. SIX1 degradation inducer results in apoptosis of PC cells. (A) Images of flowcytometry for apoptosis assay in PC cells exposed to SNS-032 for 24 h. (B) Immunofluorescence assays for cleaved-Caspase 3 in PC3 cells. Scale bar, 50 μm. (C) Apoptosis assay was performed in 22Rv1 cells exposed to si-SIX1, si-USP1 or control siRNAs for 48 h. Quantification of cell death was shown.

Table S1. PCR primers used in this study.

Gene	ACTB	GLUT1	LDHA	HK2	USP1	SIX1
Primer forward	5′-CATGTACGTTGCTATCCAGGC-3′	5′-CATCCCATGGTTCATCGTGGCTGAACT-3′	5′-ATGGCAACTCTAAGGATCA-3′	5′-GCCATCCTGCAACACTTAGGGCTTGAG-3′	5′-CCAATGAGAGCGGAAGGAGG-3′	5′-CGCGCACAATCCCTACCCATCGCC-3′
Primer reverse	5′-CTCCTTAATGTCACGCACGAT -3′	5′-GAAGTAGGTGAAGATGAAGAACAGAAC-3′	5′-GCAACTTGCAGTTCGGGC-3′	5′-GTGAGGATGTAGCTTGTAGAGGGTCCC-3′	5′-CACCAATTATATCTAGACCAAAGCC-3′	5′-CTTCCA GAGGAGAGAGTTG GTTCTG-3′
Accession number	NM_001101.5	NM_006516.4	NM_005566.4	NM_000189.5	NM_001017415.2	NM_005982.4
Amplicon size	250 bp	196 bp	400 bp	207 bp	151 bp	194 bp
Amplified target	Non-exon spanning	Non-exon spanning	Exon spanning	Exon spanning	Non-exon spanning	Exon spanning
